# Diacylglycerol *O*-Acyltransferase Type-1 Synthesizes Retinyl Esters in the Retina and Retinal Pigment Epithelium

**DOI:** 10.1371/journal.pone.0125921

**Published:** 2015-05-14

**Authors:** Joanna J. Kaylor, Roxana A. Radu, Nicholas Bischoff, Jacob Makshanoff, Jane Hu, Marcia Lloyd, Shannan Eddington, Tran Bianconi, Dean Bok, Gabriel H. Travis

**Affiliations:** 1 Jules Stein Eye Institute, University of California Los Angeles, Los Angeles, California, United States of America; 2 Department of Neurobiology, University of California Los Angeles, Los Angeles, California, United States of America; 3 Department of Biological Chemistry, University of California Los Angeles, Los Angeles, California, United States of America; University of Florida, UNITED STATES

## Abstract

Retinyl esters represent an insoluble storage form of vitamin A and are substrates for the retinoid isomerase (Rpe65) in cells of the retinal pigment epithelium (RPE). The major retinyl-ester synthase in RPE cells is lecithin:retinol acyl-transferase (LRAT). A second palmitoyl coenzyme A-dependent retinyl-ester synthase activity has been observed in RPE homogenates but the protein responsible has not been identified. Here we show that diacylglycerol *O*-acyltransferase-1 (DGAT1) is expressed in multiple cells of the retina including RPE and Müller glial cells. DGAT1 catalyzes the synthesis of retinyl esters from multiple retinol isomers with similar catalytic efficiencies. Loss of DGAT1 in *dgat1 ^-/-^* mice has no effect on retinal anatomy or the ultrastructure of photoreceptor outer-segments (OS) and RPE cells. Levels of visual chromophore in *dgat1 ^-/-^* mice were also normal. However, the normal build-up of all-*trans*-retinyl esters (all-*trans*-RE’s) in the RPE during the first hour after a deep photobleach of visual pigments in the retina was not seen in *dgat1 ^-/-^* mice. Further, total retinyl-ester synthase activity was reduced in both *dgat1 ^-/-^* retina and RPE.

## Introduction

Visual perception begins with the absorption of a photon by an opsin pigment in a rod or cone photoreceptor cell. The light absorbing chromophore in most vertebrate opsins is 11-*cis-*retinaldehyde (11-*cis-*RAL), which is photoisomerized to all-*trans-*retinaldehyde (all-*trans-*RAL), converting opsin to its active meta-II state. After briefly stimulating visual transduction, the bleached opsin decays to yield apo-opsin and free all-*trans-*RAL. Light sensitivity is restored to the apo-opsin when it combines with another 11-*cis-*RAL to form a new opsin pigment. The conversion of all-*trans-*RAL to 11-*cis-*RAL is carried out by a multi-step enzyme pathway called the visual cycle in cells of the retinal pigment epithelium (RPE). A second visual cycle for regenerating cone opsins in daylight is present in Müller glial cells of the retina [1–3]. Fatty-acyl esters of retinol play roles in the energetics of both visual cycles.

The retinoid isomerase for the canonical visual cycle in RPE cells is Rpe65 [4–6]. This enzyme uses all-*trans-*RE’s, such as all-*trans-*retinyl palmitate (all-*trans-*RP), as substrates [7, 8]. Rpe65 harnesses the energy of retinyl-ester hydrolysis to drive the endergonic conversion of all-*trans-*ROL to 11-*cis-*ROL [9]. 11-*cis-*retinyl esters (11-*cis-*RE’s) are also present in the RPE [1, 10], as a ‘pre-isomerized’ storage form of chromophore precursor. An important retinyl-ester synthase in RPE cells is LRAT, which transfers the *sn-*1 fatty acid of phosphatidylcholine (PC) to retinol [11, 12]. Although LRAT converts both all-*trans-*ROL and 11-*cis-*ROL to their cognate esters, it is more efficient as an all-*trans-*RE-synthase [13]. The acyl-coenzyme A:retinol acyl-transferases (ARAT) are a separate group of retinyl-ester synthases that use activated fatty acids such as palmitoyl coenzyme A as the acyl donors [13]. ARAT activity is present in homogenates of RPE and retina [1, 13–15]. Recently, we identified multifunctional *O*-acyltransferase (MFAT) as an 11-*cis-*specific ARAT present in Müller-glial cells of the retina [16]. MFAT is functionally coupled to retinol isomerase-2 (DES1) of the non-canonical visual cycle in Müller cells [3, 16]. However, MFAT is not present in RPE cells [16], and the protein responsible for ARAT activity in the RPE has never been identified. Besides MFAT, at least one additional ARAT activity is present in retina homogenates [16]. The protein responsible for this second ARAT activity in retina has not been identified.

DGAT1, which catalyzes synthesis of triglycerides from diacylglycerol, also has ARAT activity [17]. DGAT1 was shown to be important for retinyl-ester synthesis in the intestine, but not in adipose tissue [18]. Although DGAT1 is widely expressed in non-ocular tissues [19] and RPE cells [20], its expression in the retina has not been studied. DGAT1 is an integral-membrane protein in the endoplasmic reticulum (ER) [21]. In the current work, we evaluate the functional role of DGAT1 in the RPE and retina.

## Materials and Methods

### Ethics statement

This study was carried out in strict accordance with the recommendations in the Guide for the Care and Use of Laboratory Animals of the National Institutes of Health and the Association for Research in Vision and Ophthalmology Statement for the Use of Animals in Ophthalmic and Vision Research. The animal use protocol was approved by the University of California, Los Angeles Animal Research Committee (Permit Number: A3196-01). Euthanasia was performed by cervical dislocation in deeply anesthetized mice by intraperitoneal injections (xylazine 10mg/kg and ketamine 100mg/kg). All efforts were made to minimize discomfort, distress, pain, and injury in mice used in this study.

### General enzyme assay conditions

All experimental manipulations involving retinoids were performed under dim red light. Protein samples and solutions were kept on ice until use. Cell pellets and tissue samples were stored at −80°C, thawed on ice, and homogenized in Lysis Buffer (40 mM Tris-base, pH 8.0, 2 mM CaCl_2_, 2 mM MgCl_2_) using a glass tissue grinder (Kontes). Retinol stock solutions (5 mM) in ethanol were made fresh and stored on ice. Concentrations were determined by UV-VIS spectroscopy using reported extinction coefficients (є) [22] for all-*trans*-ROL (λ_max_ = 325 nm, є = 52,770 M^-1^cm^-1^), 11-*cis*-ROL (λ_max_ = 318 nm, є = 34,890 M^-1^cm^-1^), 13-*cis*-ROL (λ_max_ = 328 nm, є = 48,305 M^-1^cm^-1^), and palmitoyl coenzyme A(λ_max_ = 254 nm, є = 15,400 M^-1^cm^-1^). Assays were performed with continuous gentle agitation in 12x75 mm borosilicate culture tubes or 2-mL screw-cap borosilicate vials. All assays were done in triplicate. Unless otherwise stated, chemicals and solvents were purchased from Sigma-Aldrich. Protein concentrations were measured using the Micro BCA Protein Assay Kit (Pierce).

### DGAT1 enzyme kinetic analysis

Mouse DGAT1 (NM_012079) in the mammalian expression vector pcDNA3.1 (Invitrogen) was generously provided by Robert Farese (Gladstone Institute of Cardiovascular Disease, University of California, San Francisco). HEK-293T cells were grown in DMEM (Invitrogen) supplemented with 10% heat-inactivated fetal bovine serum and antibiotics (100 U/mL of penicillin G and 100 μg/mL of streptomycin) at 37°C in 5% CO_2_. HEK-293T cells were transfected (PolyFect, Qiagen) with non-recombinant pcDNA3.1 or pcDNA3.1-DGAT1. After 36–42 hours, the cells were suspended by gentle pipetting, pelleted by centrifugation at 1000 x g for five minutes, resuspended in PBS, and re-pelleted. Cell pellets were flash frozen in liquid nitrogen and stored at -80°C until use. Assays were performed in 500-μL reactions containing 40 mM Tris pH 8.0, 2 mM CaCl_2_, 2 mM MgCl_2_, and 1 mM DTT with 0.2 mg/mL cell homogenates, 0.5% BSA, 500 μM palmitoyl coenzyme A, and 0, 2, 4, 8, 16, 32, 64, 96, or 128 μM all-*trans-*ROL, 11-*cis-*ROL, or 13-*cis-*ROL substrate, as indicated. Assays were incubated for five minutes at 37°C in 2-mL glass vials with gentle agitation. The reactions were quenched, retinoids extracted, and retinyl esters quantitated by HPLC, as described below. Data were fitted to the Michaelis-Menten equation to determine *V*_max_ and *K*_M_ for each isomer using the Enzyme Kinetics Module 1.3 for SigmaPlot version 10.

### DGAT1 immunoblotting

Protein samples were harvested in PBS with protease inhibitor cocktail (Roche), homogenized, treated with 100 units of Benzonase nuclease (Sigma-Aldrich) at room temperature for one hour, and re-homogenized in the presence of 1% SDS. Samples were spun briefly in a microfuge to pellet cell debris and the supernatants collected. Protein concentrations were determined using the micro BCA-assay (Pierce). Forty-μg aliquots of each sample were loaded onto 12% Novex NuPAGE bis-tris gels (Invitrogen), run with Novex NuPAGE MOPS-SDS buffer (Invitrogen), and transferred to Immobilon-FL PVDF membranes (EMD-Millipore) using the Bio-Rad semi-dry transfer system. Blots were subsequently probed with the DGAT1 antibody (DGAT1, Abcam ab59034) in LI-COR Blocking Buffer with 0.5% donkey serum and 0.1% Tween 20 (Sigma). Secondary antibody staining and imaging were performed with LI-COR fluorescent-tagged antibodies (LI-COR) and the LI-COR Odyssey infrared imaging system.

### Bovine Müller cell primary culture

Müller cell primary cultures were prepared according to published procedures [23] with modifications to increase yield [3]. Eyes from freshly killed cattle (less than four hours) were delivered on ice from Manning Beef, LLC (Pico Rivera, CA). Eyes were washed with 50% Betadine in PBS and sectioned to yield eye cups. The retinas were dissected free of the RPE and placed in 1% antibiotic/antimycotic in Hank’s balanced salt solution (HBSS), pelleted (1500 x g for five minutes), and the supernatant discarded. The pelleted retina was resuspended in HBSS containing 5.0 mg/mL papain and incubated at 37°C for 20 minutes. Dissociated cells were rinsed and re-pelleted three times by resuspension in HBSS and centrifuged as above, to remove all traces of papain. Dissociated cells were seeded onto 60-mm plates (Corning, Cellbind) containing minimum essential medium (MEM, Invitrogen) supplemented with 10% fetal bovine serum (FBS, Gibco) and 5 mg/mL glucose (Invitrogen). Cultures were incubated at 37°C in 5% CO_2_. The media were changed by slow aspiration every 24 hours for two to three weeks until the cells were confluent. Harvesting of cells was performed as described above for 293T cells.

### Mice and genotyping

Breeder pairs of *dgat1* ^***+/-***^ mice [24] were generously provided by Robert Farese (Gladstone Institute of Cardiovascular Disease, University of California, San Francisco). Since *dgat1* ^*-/-*^ females cannot feed their young, *dgat1* ^***+/-***^ females were crossed with *dgat1* ^*-/-*^ males. Breeder pairs of *lrat* ^***-****/-*^ mice [25] were generously provided by Krzysztof Palczewski (Case Western Reserve University, Cleveland, OH). Wild-type 129S2/Sv and C57BL/6 mice were obtained from Jackson Laboratories. The genotypes of these mice at the *dgat1*, *lrat* and *rpe65* loci were determined by PCR and DNA sequencing, as previously described [24–26]. The *dgat1* ^*-/-*^ mice were homozygous for the Met450 variant in *rpe65*, while the *lrat* ^*-/-*^ mice were homozygous for the Leu450 variant. Accordingly, C57BL/6 mice (homozygous for *rpe65* Met450 variant) were used as wild-type controls for the *dgat1* ^*-/-*^ mice, while 129S2/Sv mice (homozygous for the *rpe65* Leu450 variant) were used as wild-type controls for the *lrat* ^*-/-*^ mice. Mice were raised in 12-h cyclic light at 20–40 lux. For experiments using different light conditions to observe visual cycle function, *dgat1* ^*-/-*^ mice were backcrossed eight times with 129S2/Sv mice to create homozygosity in the *dgat1* ^*-/-*^ line for the Leu450 (wild-type) variant of *rpe65*. Some mice were overnight dark-adapted followed by five minutes light exposure (~1,000 lux) and 15, 30, and 60 minutes recovery in complete darkness. These mice also received one drop of mydriatic solution in each eye and were anesthetized before bleaching under a Ganzfeld dome.

### Quantitation of gene expression by quantitative real-time PCR (qRT-PCR)

RNA from six-week-old primary cultured bovine Müller cells was prepared using the Absolutely RNA Nanoprep Kit (Agilent). Synthesis of first-strand cDNA from isolated RNA was carried out using the SuperScript III First-Strand Synthesis Supermix (Invitrogen). qRT-PCR reactions were carried out in the CFX96 Real Time C1000 Touch Thermal Cycler (BioRad) for 35 cycles under the conditions: 94°C for 20 seconds; 56°C for 30 seconds; and 72°C for 40 seconds, using iTaq Universal SYBR Green Supermix (BioRad). The primers for each gene were: actin, F: 5’CTGTCCCTGTATGCCTCTGG, R: 5’AAGGAAGGCTGGAAGAGAGC; DGAT1, F: 5’CATCCTGAATTGGTGTGTGG, R: 5’CCCACTGGAGTGATAGACTCG; MFAT, F: 5′CTTACCCGTAGAGGCACAGG, R: 5′GTAACAGGCTGAGCGTAGGG; CRALBP, F: 5’GGTCCCTGAAGAGGAACAGG, R: 5’CGAAGAGCTCTGGGTACTGC; GFAP, F: 5’CGCCAGCTACATTGAGAAGG, R: 5’ TTCCTCTCCAGATCCAGACG; DES1, F: 5’GTGGGTCTACACCGATCAGC, R: 5’GACACCGATAGGGAGATTGG. RNA levels are reported relative to actin. RNA from 8–9 week-old *dgat1* ^*-/-*^, *lrat* ^*-/-*^, 129S2/Sv and C57BL/6 mouse retinas was prepared as described above. The qRT-PCR reactions were carried out in an Opticon 2 DNA Engine (MJ Research/BioRad) using iTaq Fast SYBR Green Supermix (BioRad) (30 cycles of 94°C, 30s; 56°C, 40s; 72°C, 60 s). The primers for each gene were: GAPDH, F: 5’TGCACCACCAACTGCTTAGC, R: 5’GCCTGCTTCACCACCTTCTTG; DGAT1, F: 5’AGGTAGAAGAGGACGAGGTG, R: 5’ATTGCTGAAACCACTGTCTG; LRAT, F: 5’CACGGACCCATTTTATCCAC, R: 5’AGTCAGTCCCAACTGCTGCT. RNA levels are reported relative to GAPDH.

### Immunofluorescence of DGAT1 and cellular retinaldehyde binding protein (CRALBP) in mouse retinas

Mouse eyes from non-pigmented, wild-type (BALB/c) mice were removed after euthanization and fixed with 4% formaldehyde in 0.1 M sodium phosphate buffer (PBS) at room temperature for two hours. After anterior segments were dissected away, the eyecups were kept in fixative overnight at 4°C. Eyecups were infiltrated with 10% sucrose in 0.1 M PBS for one hour and 20% sucrose in PBS for 2 hours, and then embedded in optimal cutting temperature compound (Sakura). Ten-μm cryostat sections were cut and mounted on Superfrost Plus slides. The sections were warmed to room temperature and fixed briefly with 4% formaldehyde for five minutes and then washed with PBS three times. After the sections were bleached with Melanin Bleach Kit (Polyscience), the sections were blocked with donkey serum (0.5%, Sigma) and 1% BSA in PBS for 1 h followed by incubation with goat anti-DGAT1 in blocking buffer(1:100, Abcam) overnight at 4°C. The sections were rinsed three times with PBS with 0.1% Tween 20 and then incubated in donkey anti-goat IgG antibody conjugated with Alexafluor 488(1:500, Invitrogen) for one hour followed by rinsing. The sections were then blocked with goat serum (0.5%, Sigma) and 1% BSA for one hour and exposed to mouse anti-RLBP1 (1:75, Sigma) for one hour at room temperature. After rinsing, the sections were incubated in goat anti-mouse IgG conjugated with Alexa 568(1:500, Invitrogen). The nuclei were visualized with DAPI (Invitrogen). The sections were mounted with 5% n-propylgallate in 100% glycerol mounting medium. The images of the mouse retinal sections were captured with an Olympus FluoView FV1000 confocal laser-scanning microscope under 40X oil objective with an excitation wavelength of 488 nm and 559 nm and emission wavelength of 505–540 nm and 557–675 nm respectively.

### Light and electron microscopy of *dgat1 -/-* mouse retinas

Mice were euthanized with isoflurane and fixed by intracardiac perfusion. The primary fixative was 2% formaldehyde and 2.5% glutaraldehyde in 0.1 M sodium phosphate buffer, pH 7.4. A cautery burn marked the superior pole of the cornea for orientation before enucleation of the eye. After removal of the anterior segment, the eyecup was cut into temporal and nasal hemispheres. The nasal hemisphere was trimmed into superior and inferior quadrants. These quadrants and the hemisphere from each eye were immersed in a secondary fixative, 1% osmium tetroxide dissolved in 0.1 M sodium phosphate buffer. This was followed by dehydration in a graded series of alcohols. The quadrants were embedded in Araldite 502 (Electron Microscope Sciences). Ultrathin sections were cut on a Leica Ultracut microtome, picked up on 200 mesh copper grids, and double stained with uranium and lead salts. The sections were viewed and imaged on a Zeiss 910 electron microscope. The temporal hemisphere was embedded in an Epon-812 (Tousimis Research Corporation)/Araldite mixture. The sections were cut at 1μM thickness on the same microtome, picked up on a glass slide and stained with 1% toluidine blue in 1% sodium borate. Images were collected with a Zeiss Axiophot microscope fitted with a 40 x oil-immersion objective lens and CoolSNAP digital camera.

### ARAT enzyme assay of mouse retina and RPE homogenates

After euthanizing and enucleating two- to three-month-old *dgat1* ^*-/-*^, *lrat* ^*-/-*^, 129S2/Sv and C57BL/6 mice, the retinas and RPE were separately dissected and homogenized. These homogenates were used as enzyme sources in ARAT assays containing 0.2 mg/mL protein homogenate, 2% BSA, 50 μM all-*trans-*ROL or 11-*cis-*ROL, 150 μM palmitoyl coenzyme A, 40 mM Tris-base, pH 8.0, and 1 mM DTT. The assays were performed at 37°C for 15 minutes, mixtures were quenched by addition of methanol, and the retinoids were extracted into hexane for HPLC analysis, as described below.

### Extraction and HPLC Analysis of Retinoids

Retinoids were extracted from methanol-quenched assay mixtures by addition of 20 μL 5% SDS (0.2% SDS final concentration) followed by brief vortexing and incubation at room temperature for ten minutes to solubilize membranes. The samples were extracted twice into two mL hexane by brief vortexing and centrifugation at 3000 x g for five minutes to separate phases. The pooled hexane layers were added to 12 x 75 borosilicate test tubes and evaporated to dryness under a stream of nitrogen. Samples were dissolved in 100 μL hexane and analyzed by normal-phase HPLC in an Agilent 1100 series chromatograph equipped with a photodiode-array detector on a Supelcosil LC-Si column (4.6 × 250 mm, 5 μm) using a 0.2–10% dioxane gradient in hexane at a flow rate of two mL per minute. Spectra (210–450 nm) were acquired for all eluted peaks. The identity of each eluted peak was established by comparing the spectra and elution times with those of authentic retinoid standards. Sample peaks were quantitated by comparing peak areas to calibration curves established with retinoid standards.

### Statistical Analysis

Gene expression data for cultured Müller cells and the different mouse models were reported as average ± standard deviation (n = 4). Gene expression results were also analyzed by one-way ANOVA followed by Student-Newman-Keuls t-test to test for significance between different genes. Differences in retinoid levels between *dgat1* ^*-/-*^ versus wild-type (129S2/Sv) mice were reported as average ± standard deviation (n = 4). These data were then analyzed with a paired sample two-way ANOVA using Student-Newman-Keuls t-test to compare differences between groups of mice (wild-type or knock-out) under the same treatment condition. For *dgat1* ^*-/-*^, *lrat* ^*-/-*^, 129S2/Sv and C57BL/6 mice *in vitro* homogenate ARAT assays using all-*trans*-ROL or 11-*cis*-ROL as substrate, the results were reported as average ± standard error (n = 3). These data were further analyzed by two-way ANOVA to compare significance between the different mouse models and substrate. P-value significance was indicated by asterisks (* = p-value ≤0.05, ** = p-value ≤0.01, *** = p-value ≤0.001).

## Results

### DGAT1 is expressed in RPE and Müller-glial Cells

**Fig 1:**
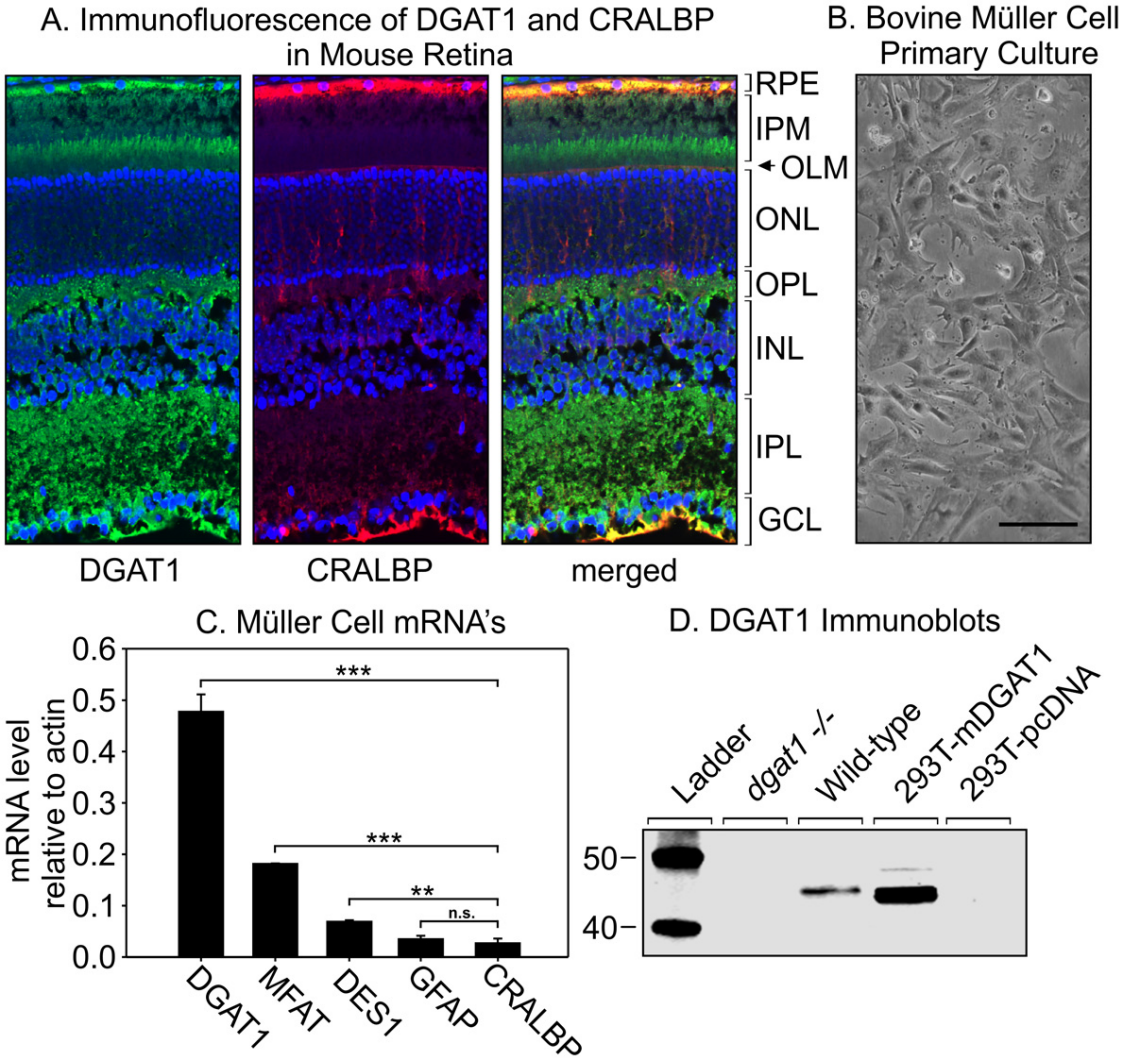
DGAT1 expression in retina and RPE. (A) Immunofluorescent analysis of DGAT1 (green) and CRALBP (red) in distal ocular sections from six-month-old BALB/c mice. Nuclei were counter-stained with DAPI (blue). The merged image shows overlapping expression of DGAT1 and CRALBP (yellow). Labels identifying retinal layers are shown to the right of the image. RPE, retinal pigment epithelium; IPM, interphotoreceptor matrix; OLM, outer limiting membrane; ONL, outer nuclear layer; OPL, outer plexiform layer; INL, inner nuclear layer; IPL, inner plexiform layer; GCL, ganglion cell layer. Müller-cell endfeet contact the vitreous within the GCL. Note expression of both DGAT1 and CRALBP in the apical microvilli of Müller cells, extending beyond the OLM into the IPM. (B) Light microscopy of twenty day old primary cultured bovine Müller-cells (scale bar = 100μm, 10X). (C) Expression of MFAT, DGAT1, CRALBP, GFAP, and DES1 mRNA’s by qRT-PCR on cDNA from primary-cultured bovine Müller-cell RNA. Levels were normalized to the actin mRNA. CRALBP, GFAP, MFAT and DES1 are positive controls for Müller-cell expression. Error bars show standard deviation of the mean for four (n = 4) independent experiments (p = <0.001, 1-way ANOVA). (D) Immunoblot of *dgat1* ^*-/-*^ and wild-type (129S2/Sv) mouse eyecup, as well as, HEK-293T cell homogenates transfected with mouse DGAT1 or non-recombinant pcDNA using antisera against mouse DGAT1.

To determine the cell types in retina that express DGAT1 we performed immunofluorescence on retina sections from wild-type (BALB/c) mice using antisera against DGAT1 and cellular retinaldehyde-binding protein (CRALBP). DGAT1 and CRALBP showed overlapping distribution in Müller and RPE cells (Fig 1A). DGAT1 immunoreactivity was present in all layers of the retina. To confirm DGAT1 expression in Müller cells, we prepared primary cultured Müller cells from bovine retinas using established procedures (Fig 1B) [3]. We determined mRNA levels for DGAT1 and the Müller-cell proteins: MFAT [16], CRALBP [27], glial fibrillary acidic protein (GFAP) [28], and DES1 [3] by qRT-PCR. All mRNA’s were normalized to the actin mRNA in the same sample. The DGAT1 mRNA was abundantly present in Müller cells relative to these other mRNA’s (Fig 1C). Immunoblotting with antisera against DGAT1 revealed bands of the predicted size (45 kDa) in homogenates of wild-type mouse eyecup and HEK-293T cells transfected with mouse DGAT1 plasmid (Fig 1D). DGAT1 immunoreactivity was not present in *dgat1* ^*-/-*^ eyecup and HEK-293T cells transfected with non-recombinant pcDNA. These results suggest that DGAT1 is expressed in multiple retinal cell-types including RPE and Müller cells where visual retinoids are processed.

### Mice lacking DGAT1 exhibit normal retinal morphology

**Fig 2:**
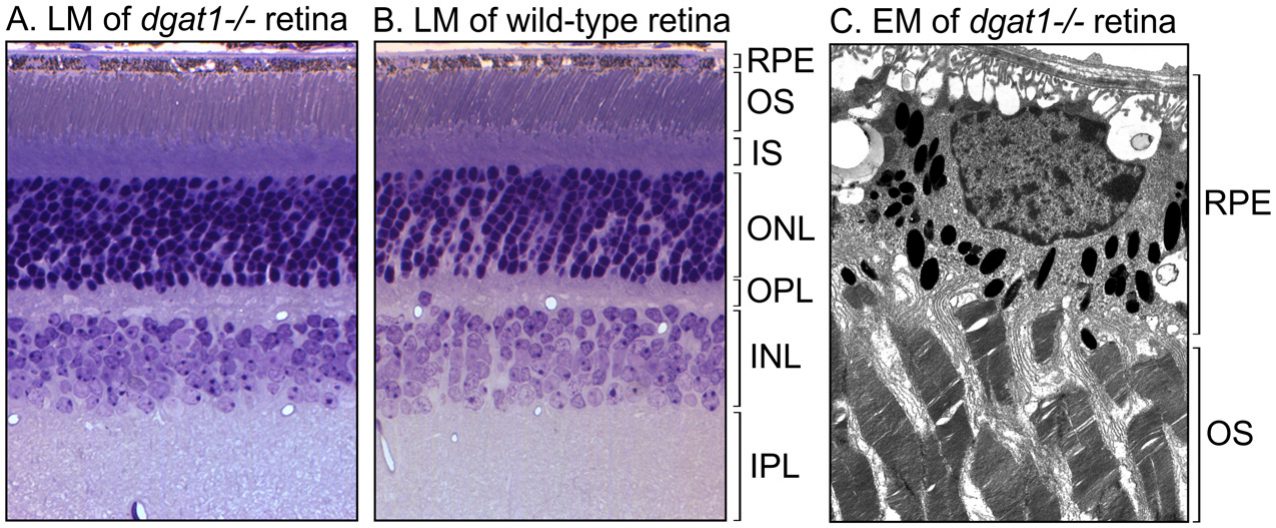
Retinal morphology in *dgat1 ^-/-^* eyes. (A) Light microscopy (LM) of a retina section from a two-month-old *dgat1* ^*-/-*^ mouse. (B) Light microscopy (LM) of a retina section from a two-month-old wild-type (129S2/Sv) mouse. Retinal layers are indicated to the right of the figure panel. (C) Electron microscopy (EM) of RPE cells, including apical microvilli, and photoreceptor outer segments (OS). RPE and photoreceptor-OS layers are indicated. RPE cells were normal. The spaces observed on the basal and lateral aspects of the cell are due to an artifact produced during perfusion of fixative through the choriocapillaris.

Loss of DGAT1 in mice causes alopecia, resistance to diet-induced obesity and the inability of *dgat1* ^*-/-*^ dams to feed their pups, all due to impaired triglyceride synthesis [24]. To study the ocular phenotype, we examined retina sections of *dgat1* ^*-/-*^ mice by light and electron microscopy. Eye size, retina morphology, and laminar organization were normal in *dgat1* ^*-/-*^ compared to wild-type mice eyes, with no evidence of photoreceptor degeneration (Fig 2A and 2B). By electron microscopy, RPE cells and photoreceptor outer segments (OS) were morphologically normal (Fig 2C). Thus, loss of DGAT1 had minimal effects on retinal anatomy.

### Loss of DGAT1 affects retinyl-ester synthesis in the eye

**Fig 3:**
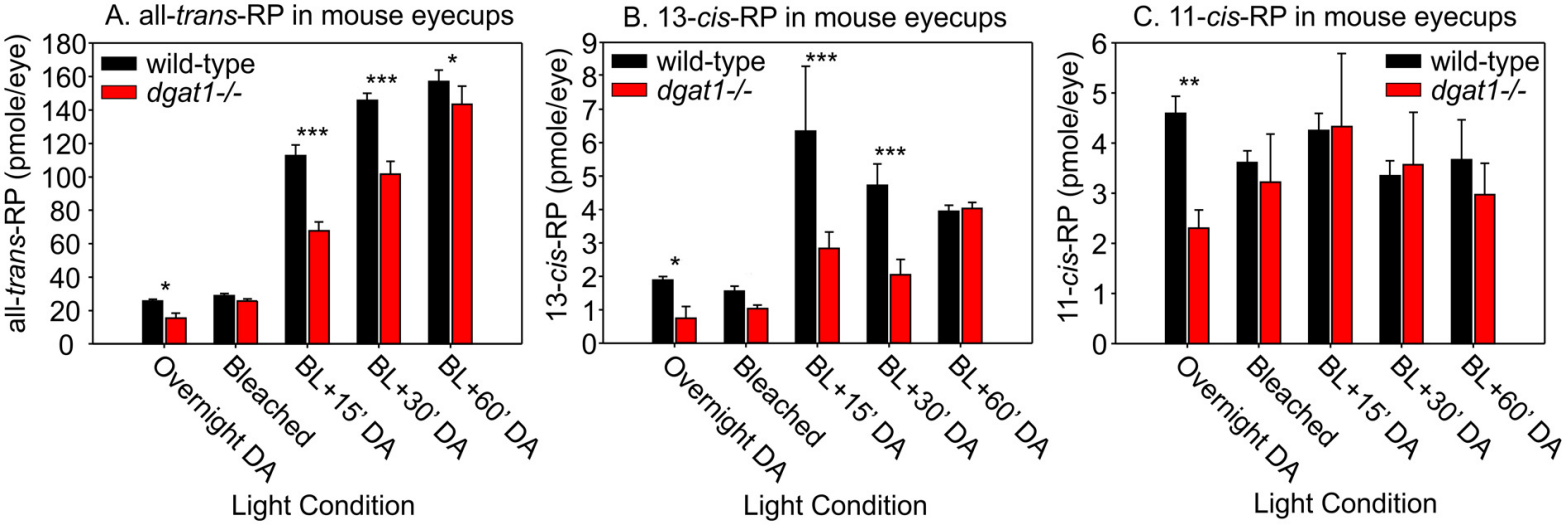
Retinyl-ester processing in *dgat1 ^-/-^* mouse eyes. Levels of (A) all-*trans-*RP, (B) 13-*cis*-RP, and (C) 11-*cis*-RP in wild-type (129S2/Sv) and *dgat1 ^-/-^* eyecups from mice that were overnight dark-adapted (DA), immediately following a deep photobleach (Bleached), and at 15, 30, or 60 minutes after returning the light-exposed animals to darkness (BL + time(mins) DA). Levels are shown as pmoles per eye. Error bars represent standard deviation of the mean for four (n = 4) eyes tested (2-way ANOVA: all-*trans*-RP, p = 0.04; 13-*cis*-RP, p = 0.08; 11-*cis*-RP, p = 0.24).

We quantitated retinyl ester levels in eyecups (retina + RPE) from wild-type (129S2/Sv) and *dgat1 ^-/-^* mice following overnight dark-adaptation, immediately following a ~60% photobleach, and at various times after returning the mice to darkness. Levels of all-*trans*-, 13-*cis*-, and 11-*cis*-retinyl palmitate were lower in overnight dark-adapted *dgat1 ^-/-^* versus wild-type eyecups (Fig 3A–3C). While 11-*cis-*retinyl palmitate (11-*cis-*RP) returned to normal after the photobleach, levels of all-*trans-*RP and 13-*cis-*RP remained lower in *dgat1 ^-/-^* versus wild-type eyecups at 15 and 30 minutes post-bleach, returning to normal by one hour post-bleach (Fig 3A and 3B).

### Estimated substrate kinetics of palmitoyl coenzyme A-dependent retinyl-ester synthase activity of DGAT1

**Fig 4:**
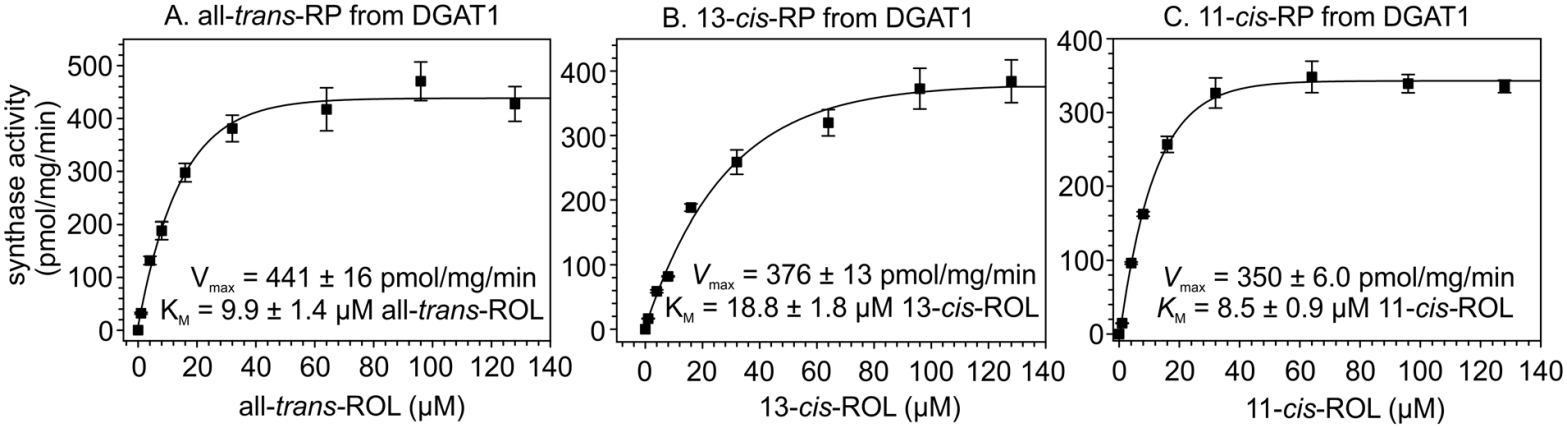
Kinetic analysis of DGAT1 activity with different retinol isomers. Homogenates of 293T cells expressing DGAT1 were assayed for palmitoyl coenzyme A-dependent retinyl-ester synthase activities using (A) all-*trans*-ROL, (B) 13-*cis*-ROL, or (C) 11-*cis*-ROL as substrate at the indicated concentrations. Non-linear fitting of these data with the SigmaPlot Kinetics module yielded *V*_max_ and *K*_M_ values for synthesis of each retinyl-ester isomer from its cognate retinol. Note the similar *V*_max_ values for each isomer. Activities are expressed as pmoles per mg total protein per minute. Error bars show standard error of the mean for three (n = 3) samples independently tested. R-squared values were 0.97, 0.99, and 0.99 for A, B, and C, respectively.

Because the loss of DGAT1 caused changes in the levels of several retinyl-ester isomers, we determined the kinetics of retinyl ester synthesis by DGAT1 for the different retinol isomers. Here, we used homogenates of HEK-293T cells expressing mouse DGAT1 as an enzyme source to obtain an estimate of substrate activity and specificity. For each retinol isomer we determined the initial synthesis rate (*V*_0_) of its cognate retinyl ester by DGAT1 at various substrate concentrations (Fig 4A–4C). To avoid measuring the background retinyl-ester synthase activity in 293T cells, we subtracted retinyl esters synthesized by non-transfected 293T-cell homogenates from those produced by homogenates of DGAT1-transfected cells. Michaelis-Menten analysis of these data yielded the maximum turnover-rate (*V*_max_) and Michaelis constant (*K*_M_) for each isomer. DGAT1 synthesized all-*trans-*RP, 13-*cis-*RP and 11-*cis-*RP at similar rates (Fig 4A–4C). The *K*_M_ of DGAT1 for all-*trans-*ROL was approximately half that for 13-*cis*-ROL, indicating a greater specificity for all-*trans*-ROL substrate (Fig 4A and 4B). DGAT1 is hence an efficient synthase of all-*trans-*retinyl esters, which is reflected by the significant loss of all-*trans*-RP levels during recovery in *dgat1 ^-/-^* mice (Fig 3A). DGAT1 shows no preferential substrate specificity (low *K*_M_) for synthesis of 11-*cis*-RP over all-*trans*-RP (Fig 4A and 4C). Based on these data, DGAT1 appears to be a non-stereospecific retinyl-ester synthase.

### Loss of DGAT1 does not affect regeneration of visual chromophore

**Fig 5:**
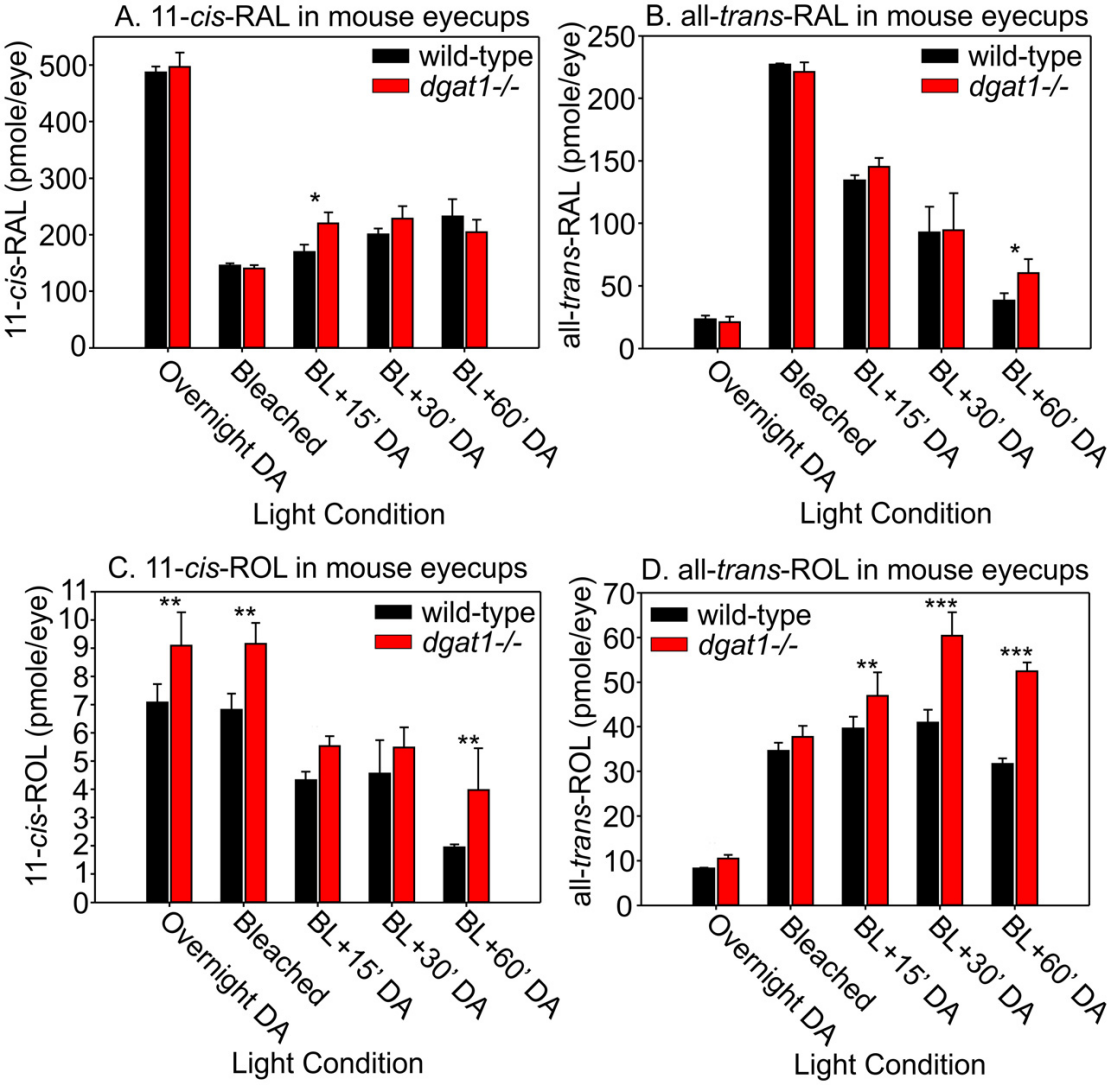
Retinal and Retinol processing in *dgat1 ^-/-^* mouse eyes. Levels of (A) 11-*cis*-RAL, (B) all-*trans*-RAL, (C) 11-*cis*-ROL, and (D) all-*trans*-ROL in wild-type (129S2/Sv) and *dgat1 ^-/-^* eyecups from mice that were overnight dark-adapted (DA), immediately following a deep photobleach (BL), and at 15, 30, or 60 minutes after returning the light-exposed animals to darkness (BL + time(mins) DA). Levels are shown as pmoles per eye. Error bars represent standard deviation of the mean for four (n = 4) eyes tested (2-way ANOVA: 11-*cis*-RAL, p = 0.46; all-*trans*-RAL, p = 0.36; 11-*cis*-ROL, p = 0.31; all-*trans*-ROL, p = 0.08).

Levels of 11-*cis*-RAL in mouse eyecups were unaffected by loss of DGAT1 (Fig 5A), suggesting that all-*trans*-RE’s produced by DGAT1 do not contribute to chromophore regeneration. Levels of all-*trans-*RAL, produced by bleaching of opsin pigments, were similar in wild-type and *dgat1 ^-/-^* eyecups (Fig 5B). Pre and post-bleach 11-*cis*-retinol levels were slightly elevated in DGAT1 knockout mice (Fig 5C). This was also true for all-*trans*-retinol levels before bleach and during recovery in the dark (Fig 5D). Elevated 11-*cis*-ROL and all-*trans*-ROL in *dgat1 ^-/-^* mice eyes are probably due to loss of retinyl-ester synthase activity.

### Loss of DGAT1 affects total acyl-coenzyme A:retinol acyl-transferase (ARAT) activity in the eye

**Fig 6:**
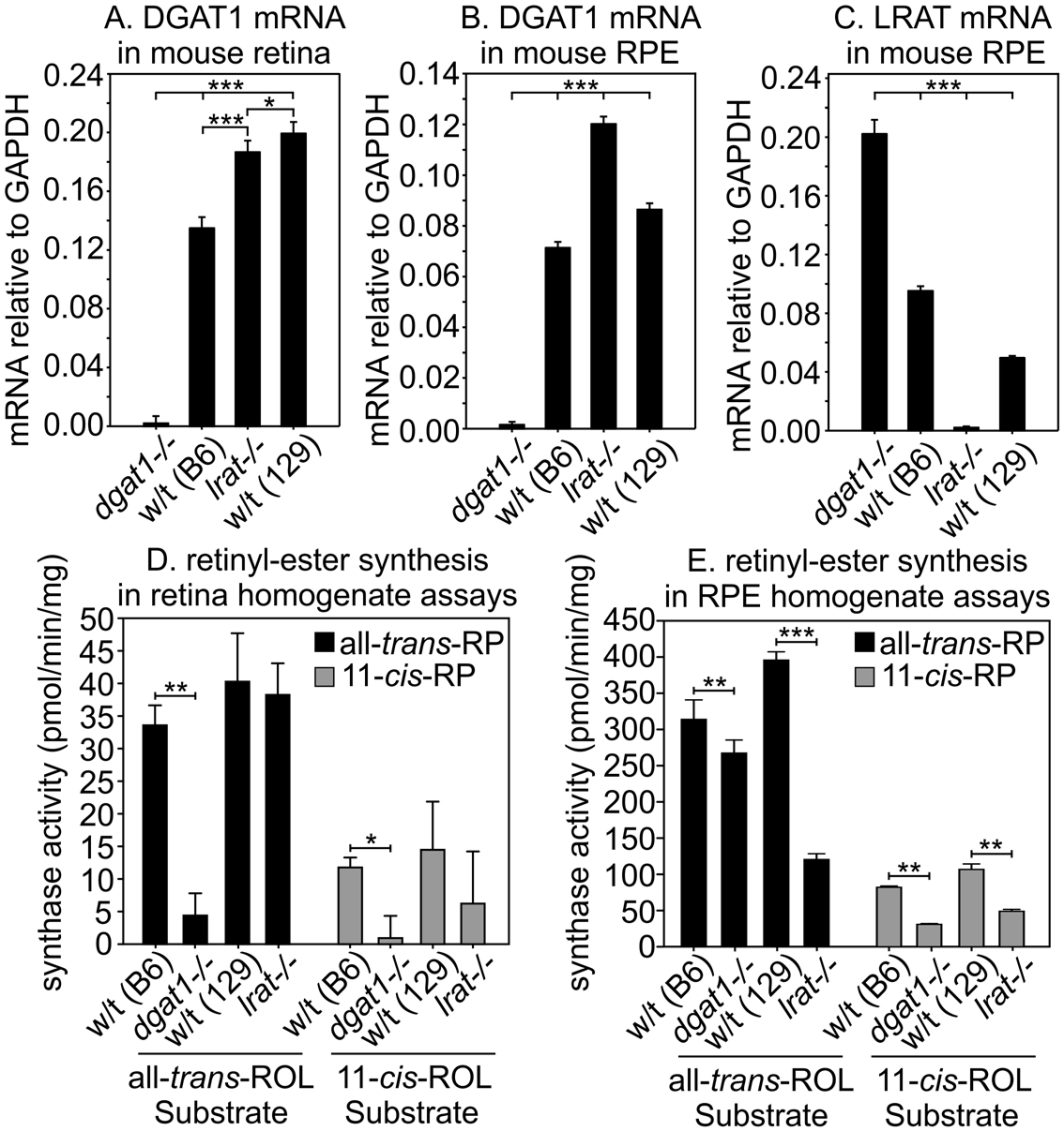
Retinyl-ester processing in *dgat1 ^-/-^* and *lrat^-/-^* mouse eyes. (A) Levels of the DGAT1 mRNA by qRT-PCR in retina samples from mice of the indicated genotypes. Levels were normalized to the GAPDH mRNA. (B) Levels of the DGAT1 mRNA by qRT-PCR in RPE samples from mice of the indicated genotypes. Levels were normalized to the GAPDH mRNA. (C) Levels of the LRAT mRNA by qRT-PCR in RPE samples from mice of the indicated genotypes. Levels were normalized to the GAPDH mRNA. Error bars for (A), (B), and (C) represent standard deviation of the mean for four (n = 4) samples of mRNA tested (p = <0.001, 1-way ANOVA for (A), (B), and (C)). (D) Retinyl-ester synthase activities of wild-type (C57BL/6 or 129S2/Sv), *dgat1 ^-/-^* and *lrat^-/-^* mouse retina homogenates using all-*trans-*ROL or 11-*cis-*ROL as substrate. Activities are shown as pmoles all-*trans-*RP or 11-*cis-*RP per minute per mg protein (2-way ANOVA: (D), p = 0.04, all-*trans*-ROL; p = 0.41, 11-*cis*-ROL). (E) Retinyl-ester synthase activities of wild-type (C57BL/6 or 129S2/Sv), *dgat1* ^*-/-*^ and *lrat^-/-^* mouse RPE homogenates using the same substrates as in (D) (2-way ANOVA: (D), p = <0.001, all-*trans*-ROL; p = <0.001, 11-*cis*-ROL). Activities are shown as pmoles all-*trans-*RP or 11-*cis-*RP per minute per mg protein. Error bars for (D) and (E) represent the standard error of the mean for three (n = 3) individual ARAT assays on the mouse type and tissue specified

Besides MFAT, at least one additional ARAT activity is present in retina homogenates [16]. The protein responsible for this second ARAT activity in the eye has not been identified. To determine the role of DGAT1 on total retinyl-ester synthesis in mouse eyes, we compared ARAT activities in mice lacking LRAT or DGAT1. First, we determined levels of the DGAT1 mRNA in retina and RPE samples from wild-type (129S2/Sv and C57BL/6), *dgat1^-/-^*, or *lrat^-/-^* mice by quantitative real-time PCR (qRT-PCR), normalizing to glyceraldehyde 3-phosphate dehydrogenase (GAPDH) in the same samples. DGAT1 mRNA levels were similar in wild-type and *lrat^-/-^* retinas (Fig 6A). The DGAT1 mRNA was 1.25-fold more abundant in *lrat^-/-^* versus wild-type RPE (Fig 6B), possibly representing compensatory over-expression in the absence of LRAT. Similarly, the LRAT mRNA was two-fold more abundant in *dgat1^-/-^* versus wild-type RPE (Fig 6C), again suggesting compensatory up-regulation in the absence of DGAT1. We then prepared retina and RPE homogenates from *dgat1 ^-/-^* and *lrat^-/-^* mice and their wild-type background strains, C57BL/6 and 129S2/Sv, respectively. We assayed these homogenates for ARAT activity in the presence of all-*trans-*ROL or 11-*cis-*ROL and palmitoyl coenzyme A. These assay conditions are also permissive for LRAT activity [13], as the homogenates contain PC, the acyl donor for LRAT. In retina homogenates, loss of DGAT1 caused profound suppression of all-*trans* and 11-*cis-*RP synthase activities, while loss of LRAT had little effect (Fig 6D). This observation is in agreement with non-expression of LRAT in the retina [14]. In contrast, loss of DGAT1 resulted in 15% loss of all-*trans-*RP synthase and 63% loss of 11-*cis-*RP synthase activities in the RPE, which does express LRAT (Fig 6E). Retinyl-ester synthase activities were approximately 10-fold higher in RPE versus retina homogenates (Fig 6D and 6E), consistent with the much higher retinyl esters in mouse RPE versus retinas [1]. The RPE retained 30% of all-*trans-*RP-synthase and 45% of 11-*cis-*RP-synthase activities in *lrat^-/-^* mice (Fig 6E). These results suggest that DGAT1 contributes to ARAT activities in both the retina and RPE.

## Discussion

Besides LRAT, RPE homogenates possess a palmitoyl coenzyme A-dependent retinyl-ester synthase activity that fatty-acylates all-*trans-* and 11-*cis-*ROL [13]. Results presented here suggest that DGAT1 is responsible for much of this ARAT activity. Loss of DGAT1 in *dgat1* ^*-/-*^ mice had no effect on retinal histology (Fig 2A and 2B) or the ultrastructure of photoreceptor OS and RPE cells (Fig 2C). Biochemically, loss of DGAT1 in *dgat1* ^*-/-*^ mice reduced all-*trans-*RE-synthase activity by ~15% and 11-*cis-*RE-synthase activity by ~65% in the RPE (Fig 6E). In contrast, loss of LRAT in *lrat^-/-^* mice reduced all-*trans-*RE-synthase activity by ~70% and 11-*cis-*RE-synthase activity by ~55% in the RPE (Fig 6E). Thus, DGAT1 may contribute up to 30% of the retinyl-ester synthase activity (ARAT) in the RPE.

Despite presumed expression of DGAT1, *lrat* ^***-****/-*^ mice contain only ‘trace’ retinyl esters in the RPE [25]. Uptake of retinol from blood into the RPE is mediated by the STRA6 receptor for retinol-binding protein, and is driven by mass action through subsequent esterification of retinol [29]. The concentration of retinol in mouse serum is approximately one micromolar [30]. This value is close to the *K*_M_ of LRAT (0.24 μM, [31]) but nearly ten-fold lower than the estimated *K*_M_ of DGAT1 for all-*trans-*ROL (Fig 4A). Thus, DGAT1 is far less effective than LRAT at driving retinol uptake from blood. How might RPE cells benefit by expressing a second retinyl-ester synthase with lower substrate sensitivity? During the first hour of recovery in the dark following a deep photobleach, all-*trans-*RE’s accumulate in the RPE six-fold over dark-adapted levels [32, 33] (Fig 3A). This results from the high levels of all-*trans-*ROL released by the bleached photoreceptors and the declining demand for synthesis of new chromophore after the return to darkness. The normal build-up of all-*trans-*RP during post-bleach recovery was delayed in *dgat1* ^*-/-*^ mice (Fig 3A). This observation suggests that the retinyl-ester synthase activity of DGAT1 becomes physiologically relevant under conditions of high all-*trans-*ROL. By expressing two retinyl-ester synthases with staggered *K*_M_’s, the RPE gains additional ester-synthase capacity when required, without driving all-*trans-*ROL to very low levels, or causing continuous accumulation of all-*trans-*RE’s.

The role of DGAT1 as a retinyl-ester synthase in the retina is less clear. While retinyl esters are present in the RPE of all animal species studied [1, 15], only cone-dominant species contain significant retinyl esters in the retina, and they are predominantly 11-*cis-*RE’s [1, 3, 10]. Loss of DGAT1 in *dgat1* ^*-/-*^ retina homogenates resulted in a further 10-fold reduction in total all-*trans-*RE and 11-*cis-*RE synthase activities under *in vitro* conditions (Fig 6D). In contrast, we observed no significant difference in total all-*trans-*RE or 11-*cis-*RE synthase activities in wild-type versus *lrat^-/-^* retina homogenates (Fig 6D), consistent with non-expression of LRAT in retinas [14]. These results suggest that DGAT1 is a significant retinyl-ester synthase in mouse retinas. Addition of all-*trans*-ROL substrate to retinas from cone-dominant chickens or ground squirrels results in its conversion to 11-*cis*-RE’s, in contrast to rod-dominant retinas, which make lower amounts of all-*trans*-RE’s [1]. This observation implies the existence of an 11-*cis-*specific retinyl-ester synthase in Müller cells that acts cooperatively with isomerase-2 (dihydroceramide desaturase 1 or DES1) of the non-canonical visual cycle [3]. DGAT1 is not this synthase, despite being expressed in Müller cells (Fig 1B and 1C), since it esterifies retinol isomers with similar catalytic efficiency (Fig 4). Recently, multifunctional *O-*acyltransferase (MFAT) was shown to be an 11-*cis-*specific retinyl-ester synthase functionally coupled to DES1 in Müller cells [16].

Retinyl esters are contained within two compartments of RPE cells: ER membranes and lipid droplets [34]. Rpe65 associates with the ER [35], but was not found in association with lipid droplets [34]. It could be that DGAT1 is responsible solely for synthesis of retinyl esters in lipid droplets. Loss of DGAT1 would then have little effect on chromophore synthesis before the ER pools of retinyl esters are depleted. This could explain the similar dynamics of chromophore synthesis in wild type and *dgat1* ^*-/-*^ mice (Fig 5A). It should be noted that DGAT1 plays a role in the storage of fatty-acyl CoA’s and is a therapeutic target for treatment of obesity [36]. Based on the studies presented here, despite its involvement in retinoid metabolism, inhibition of DGAT1 activity *in vivo* may not affect vision, especially with regards to chromophore regeneration.

In summary, DGAT1 functions as a palmitoyl coenzyme A-dependent retinyl-ester synthase in cells of the RPE and retina including Müller glial cells. In the RPE, DGAT1 may function together with LRAT to synthesize all-*trans-*RE’s when the concentration of all-*trans-*ROL is high, such as shortly following a deep photobleach. DGAT1 appears to serve as a non-stereospecific retinyl-ester synthase in the RPE and Müller cells of the retina.
